# Prior Information Shapes Perceptual Confidence

**DOI:** 10.5334/joc.417

**Published:** 2025-01-07

**Authors:** Luca Tarasi, Margherita Covelli, Chiara Tabarelli de Fatis, Vincenzo Romei

**Affiliations:** 1 Dipartimento di Psicologia, Universitàdi Bologna and Centro studi e ricerche in Neuroscienze Cognitive, Universitàdi Bologna, Cesena, Italy; 2 Universidad Antonio de Nebrija, Madrid, Spain

**Keywords:** Predictive coding, Perceptual decision-making, Perceptual Confidence, Metacognition, Signal Detection Theory

## Abstract

Decisional confidence refers to the subjective evaluation of the accuracy of a decision based on sensory information. While these judgments are typically grounded in the strength of evidence leading to a decision, they are also subjected to influence from top-down factors such as prior expectations. Previous research has highlighted the impact of prior information on decision parameters such as reaction times and decision criteria placement. However, a comprehensive understanding of how prior information shapes confidence ratings is still lacking. In this study, we manipulate prior knowledge by inducing varying levels of target probability expectation (low: 33%, random: 50%, high: 67%) in a perceptual detection task. In each trial both type-1 (detection) and type-2 (confidence) responses were recorded. First, we replicate previous findings, demonstrating that decisional priors impact decision criteria but not task sensitivity. Secondly, we reveal the strong effect that prior expectations exert on type-2 decisions, with this influence being moderated by a congruency effect between the given prior, the actual stimulus presented, and the provided response. Moreover, we find that confidence is higher in correct compared to incorrect responses, with low-probability trials leading to higher confidence judgments in correct choices compared to random and liberal trials. Finally, we unveil that prior-dependent modulation rates in criterion and confidence were positively associated. These results underscore the intricate interplay between prior expectations, decision-making, and confidence levels, demonstrating that what we perceive is not solely a data-driven phenomenon but can be already shaped by the available information about the state of the world.

## Introduction

In our day-to-day existence, we frequently encounter scenarios that require us to make decisions, selecting from various options that represent different possible outcomes. To aid in this decision-making process, perceptual input received through our senses enables us to accumulate evidence supporting one alternative over another (Gold & Shadlen, 2007). Decisions concerning the state of the world, such as determining whether a particular event has occurred or not, are termed stimulus-conditioned responses or Type 1 decisions. However, in the realm of perceptual decision-making, another decisional dimension (Type 2 decisions) concerns the observer’s ability to assess the accuracy of their decisions, commonly referred to as decision confidence (Galvin et al., 2003; Mamassian, 2016), which significantly influences our decision-making capability (Braun et al., 2018). While type-1 sensitivity and visual confidence are often related, they can be double-dissociated both in terms of performance and their underlying neural foundations (Di Gregorio et al., 2022; Di Luzio et al., 2022; Maniscalco et al., 2016; Sahraie et al., 1998; Trajkovic et al., 2024).

However, when engaging in perceptual decision-making, it is essential to extend beyond the sensory evidence at hand and incorporate knowledge about the potential world states, commonly referred to as *priors*. Various studies have demonstrated that priors significantly influence the amount of evidence required to favor one alternative over another, thereby leading to a shift in the decision-making parameters such as reaction time and decisional criterion and facilitating prior-congruent responses (Bang & Rahnev, 2017; De Lange et al., 2013; Mulder et al., 2012; Wyart et al., 2012; Zhou et al., 2021; Tarasi et al., 2024). The effect of priors on decisional bias has been clearly demonstrated in a study employing a detection task, where a cue indicated the probability of target presence in the upcoming trial (Tarasi, di Pellegrino, et al., 2022). Participants adjusted their decisional criteria based on the probability of target presence, demonstrating a more liberal criterion in the presence of a high target probability and a more conservative one when the target probability was low. However, this modulation was not observed in sensitivity (d-prime), indicating an absence of the effect of priors on this key perceptual component.

Overall, previous studies converge in establishing a role of prior information in shaping type 1 decisions. However, there is still a lack of a comprehensive understanding regarding its impact on confidence judgments, with only a small number of experiments focusing on investigating this aspect (Constant et al., 2023; Locke et al., 2020; Sherman et al., 2015). Maniscalco and Lau’s (2014) work within the framework of Signal Detection Theory (SDT) and its extensions provides a theoretical foundation for exploring this relationship. They suggest that confidence judgments (Type 2 tasks) arise from the same internal processes that guide stimulus classification (Type 1 tasks), with stimulus and response types acting as moderators that shape both decisions and confidence by influencing internal signal distributions and decision criteria.

To bridge this gap, we employed a probabilistic detection task in which expectations-like information was presented in a trial-by-trial fashion, informing about the probability of target presence in the upcoming trial. Participants were required to provide both Type 1 (i.e., stimulus present or absent) and Type 2 responses (i.e., decision confidence recorded through a Likert scale 1-to-4). This enabled the investigation, on a trial-by-trial basis, of the interplay between the provided prior, the actual stimulus presented, participants’ response, and the resulting confidence judgment. Additionally, it provides the opportunity for a comparative analysis, exploring the relationship between prior-driven criterion and confidence modulation.

We hypothesized that 1) priors can indeed exert an influence on type-2 decisions, as these judgments represent the subjective perception of decisional accuracy that can be influenced by top-down factors (Rahnev et al., 2011); 2) the relationship between prior and confidence is moderated by the congruence between the provided priors and the subsequent stimuli presented, or the reported responses and 3) the prior-driven criterion and confidence modulations are associated.

## Materials and Methods

### Experimental Design

**Figure 1:**
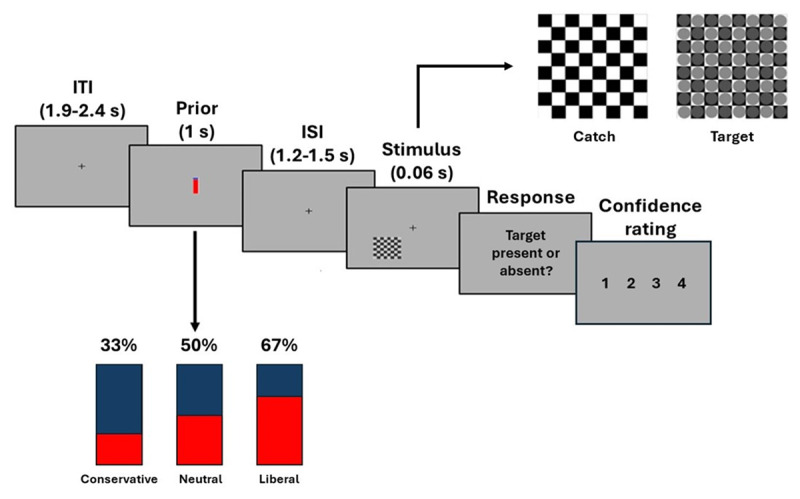
Experimental design. Behavioural data were collected during a simple visual detection task. Each trial started with a fixation cross, followed by the appearance of a probabilistic cue at the centre of the screen. Subsequently, a checkerboard, either containing or not grey circles at the titrated contrast level, appeared at the bottom left corner of the monitor for a duration of 0.06 seconds. Following stimulus presentation, two screens were displayed, the first asking for type 1 (target detection) and the second for type 2 (confidence rating) responses respectively. The presented cues took the form of a blue bar, filled with different levels of red ink in the proportion of 33, 50 and 67% of the bar. The degree of red filling of the blue bar was related with the probability of target presence, with 33% indicating low probability and 67% high probability of target, corresponding to conservative and liberal conditions, respectively while the half-filled (50%) bar representing the random target probability occurrence, being thus uninformative of target presence vs. absence.

Seventy-five healthy participants (39 female, age range 18–35) signed a written informed consent prior to take part in the study, which was conducted in accordance with the Declaration of Helsinki and approved by the Bioethics Committee of the University of Bologna (protocol code 201723, approved on 26 August 2021). The sample size was determined to enable comparison with a previous study (Tarasi, di Pellegrino, et al., 2022) investigating the relationship between prior information and type-1 decisions. Participants performed a detection task while providing confidence judgments regarding their decision in the perceptual task (Figure 1). In the first phase, participants were presented with visual stimuli (checkerboards which could contain grey circles – target trials – or not – catch trials) and underwent an adaptive titration procedure akin to the one employed in Tarasi and Romei (2023) to determine the contrast of the grey circles at which detection accuracy was at ~70%. The second phase comprised 6 blocks of 90 trials each. Each trial began with the presentation of a cue indicating the probability of target presence (low: 33%, random: 50% and high probability: 67%). Participants were explicitly informed that the actual probability of target presentation matched the prior information. Following a variable delay, a checkerboard containing (or not) grey circles appeared, and participants were required to give two consecutive responses: the first, concerning the presence or absence of the grey circles, the second, concerning the confidence rating for their choice on a Likert scale 1-to-4 with 1 indicating low confidence and 4 high confidence. A more detailed description of the experimental procedure can be found in Supplementary File 1.

### Signal detection theory (SDT)

We computed the SDT measures sensitivity (*d*′) and criterion (*c)* (Green & Swets, 1966). *d*′ quantifies a participant’s stimulus sensitivity, while *c* quantifies decisional bias. To evaluate the effect of the probabilistic cue on sensitivity and criterion, we computed *d*′ and *c* separately for trials preceded by low, high, or random probability cues. To statistically investigate a cue-related effect, *d*′ and *c* were subjected to a repeated-measures ANOVA with the cue type as the within-factor (3 levels: high, low, or random probability).

### Confidence

To evaluate the influence of the probabilistic cue on confidence ratings, we conducted a repeated-measures ANOVA, with cue type as the within-factor (3 levels: high, low, or random probability). To assess whether prior information affected confidence ratings differently for correct and incorrect responses, we conducted a repeated-measures ANOVA with cue type and correctness (2 levels: correct, incorrect response) as within-factors. Additionally, to examine whether the impact of prior information on confidence varied according to the stimulus (between target-present and target-absent trials) and to the response (between target-present and target-absent responses), two ANOVAs were conducted. Both ANOVAs included cue type as a within-subjects variable. In one analysis, the stimulus was considered as a within-factor (2 levels: present or absent), whereas in the second analysis the subject’s response was the within-factor (2 levels: present or absent).

### Meta-d′

Metacognitive performance was assessed using the meta-d′ model, which evaluates the effectiveness of confidence ratings in distinguishing between correct and incorrect responses, independent of perceptual sensitivity. Following Maniscalco and Lau’s (2012) method, meta-d′ was computed using the fit_meta_d_MLE function in MATLAB. Given the understanding that meta-d is influenced by type 1 decision sensitivity, we employed the metad/d metric, which adjusts for baseline sensitivity differences when assessing individual metacognitive ability. To evaluate how variations in prior probabilities affect Meta-d′/d, we performed a repeated-measures ANOVA, with cue type as the within-subjects factor (3 levels: high, low, or random probability).

### Correlations

To investigate the relationship between prior-driven criterion and confidence shift, we conducted several correlation analyses. Initially, we computed Pearson correlation between the mean criterion values and the mean confidence ratings separately for trials preceded by high, low, and neutral probability cue. Furthermore, we conducted several correlation analyses to explore the relationship between the modulation of criterion and the modulation of confidence induced by the probabilistic cue. We took the difference between the criterion adopted in low and high probability trials (*Δ criterion* = crit_low_ – crit_high_) as a proxy of criterion modulation (for a similar approach, see De Lange et al., 2013). Regarding confidence, we took the difference between confidence ratings in prior congruent vs. prior incongruent trials (*Δ* confidence = conf_congruent_ – conf_incongruent_) relative to both the stimulus and the response. Thus, we derived four distinct measures of confidence shift: concerning the stimulus, we computed *Δ* confidence separately for target-present and target-absent trials. Similarly, regarding the response, we computed *Δ* confidence separately for stimulus-present and stimulus-absent responses. A more detailed description of the computations for these indices are included in Supplementary File 1.

## Results

### Decision criterion, but not sensitivity, is modulated by prior information

We computed the signal detection theory indices *d*′ (sensitivity) and *c* (criterion) (Green & Swets, 1966) separately for trials preceded by low, high, and random probability cues. As expected, the repeated-measures ANOVA found an impact of probabilistic cue on the decision criterion (F_2,148_ = 84.49; p < 0.001). Specifically, the criterion adopted in trials preceded by high probability cue (crit_high_ = –0.03 ± 0.06) was more liberal relative to trials preceded by random (crit_random_ = 0.42 ± 0.05; t_74_ = 8.00) and low probability cues (crit_low_ = 0.70 ± 0.06; t_74_ = 12.87; all p < 0.001), while criterion was more conservative in trials preceded by low relative to random probability cue (t_74_ = 4.88; p < 0.001). In contrast, the cue had no effect on sensitivity (F_2,148_ = 1.38; p = 0.254).

### Perceptual confidence increases in conservative trials

To assess whether confidence was modulated by prior information, we compared confidence ratings between trials preceded by high, low, and random probability cues. The ANOVA revealed a significant effect of the cue (F_2,148_ = 9.99; p < 0.001). Specifically, confidence ratings in conservative trials (i.e., when the low probability cue was presented) were higher (conf_low_ = 3.04 ± 0.04) relative to liberal trials (i.e., when the high probability cue was presented; conf_high_ = 2.96 ± 0.04; t_74_ = 3.49; p = 0.001) and random trials (conf_random_ = 2.94 ± 0.05; t_74_ = 4.17; p < 0.001). However, the same ANOVA conducted on Meta-d′ did not show a significant effect (F_2,148_ = 1.608; p = 0.204), indicating that priors do not significantly influence Meta-d′ values.

**Figure 2:**
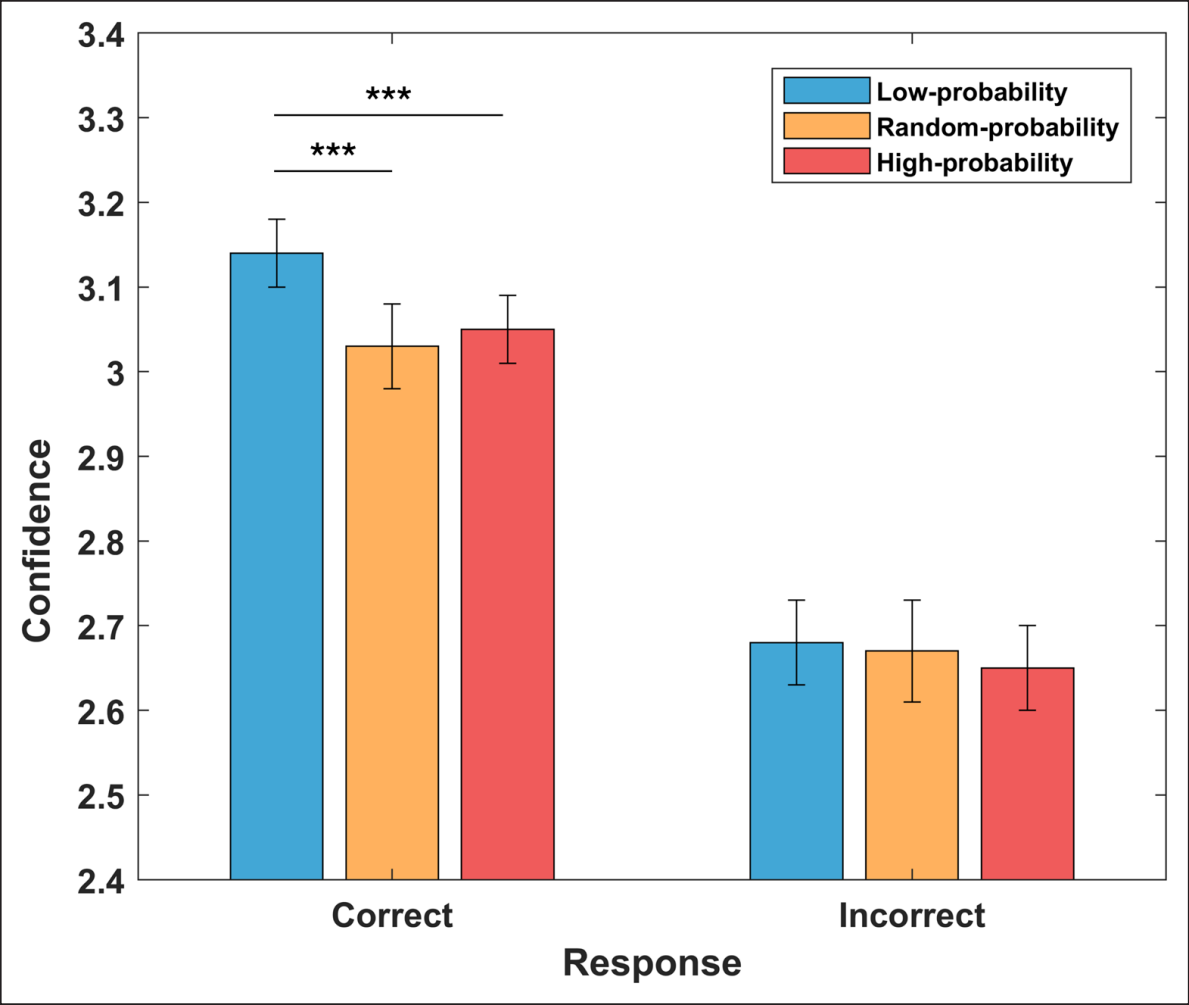
Interaction between prior information and response correctness in shaping confidence levels. Confidence levels were influenced by the accuracy of the responses, showing greater levels following correct relative to incorrect responses. Moreover, no difference between probability trials were observed for incorrect responses, while higher confidence was observed on low-probability trials compared to random and high probability trials for correct choices.

We then examined whether prior influence on confidence ratings was moderated by the correctness of participants’ responses (Figure 2). The ANOVA showed a significant interaction between prior information and correctness (F_2,148_ = 4.34; p = 0.015). Post-hoc analyses revealed that confidence is higher for correct relative to incorrect responses (correct vs. incorrect low-probability 3.14 ± 0.04 vs 2.68 ± 0.05; t_74_ = 14.48; correct vs incorrect high-probability 3.05 ± 0.04 vs 2.65 ± 0.05; t_74_ = 10.54; correct vs. incorrect random-probability 3.03 ± 0.05 vs = 2.67 ± 0.06; t_74_ = 9.44; all p < 0.001). Crucially, confidence in the conservative condition was higher relative to both liberal (t_74_ = 3.59; p < 0.001) and random conditions (t_74_ = 4.95; p < 0.001), but only for correct responses.

### Congruency between stimulus/response and prior information enhances perceptual confidence

**Figure 3:**
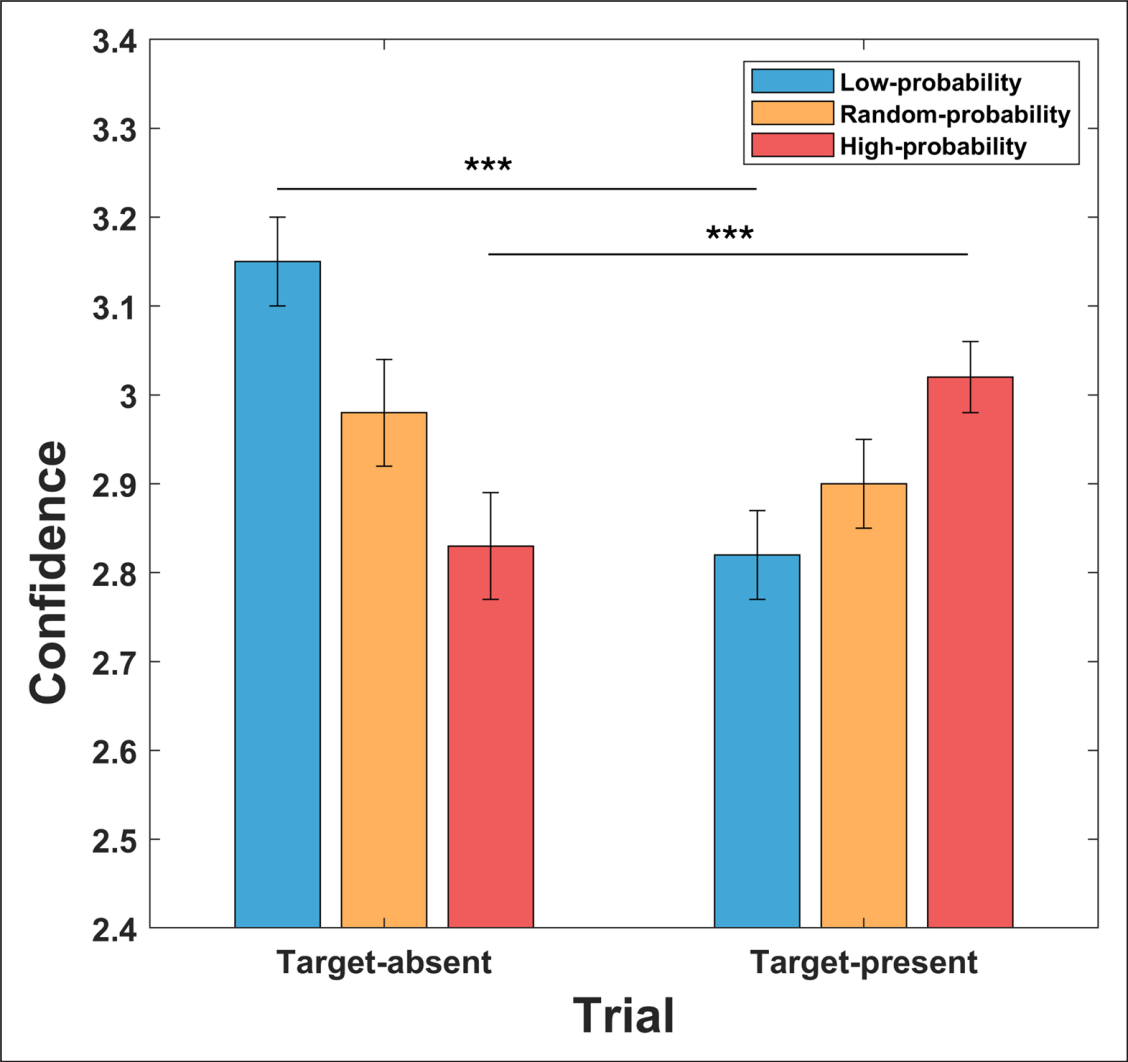
Congruency effect between prior information and stimulus presented. Prior information influenced confidence levels based on the congruence between target probability and subsequent target presence. After catch trials, higher confidence was observed when the low-probability cue (33%) was presented compared to the high-probability one, whereas in target trials, the reverse trend was evident, with higher confidence linked to the high-probability cue (67%). Following the presentation of the random-probability cue (50%), no distinction in confidence levels between target and catch trials was observed.

We evaluated the effect of prior information on confidence separately for trials where the target was present (target trials) vs. absent (catch trials) (Figure 3). The ANOVA revealed a significant interaction between prior information and stimulus presentation (F_2,148_ = 70.46; p < 0.001). After the appearance of the high probability cue, confidence was higher when the target was actually presented (3.02 ± 0.04) relative to catch trials (2.83 ± 0.06; t_74_ = –4.23; p < 0.001), while in the low probability condition, the opposite pattern emerged, with higher confidence ratings for catch trials (3.15 ± 0.05) compared to target trials (2.82 ± 0.05; t_74_ = 6.68; p < 0.001). A nonsignificant trend in the same direction as the low probability condition was found for trials preceded by the random probability cue (target vs catch = 2.90 ± 0.05 vs 2.98 ± 0.06; t_74_ = 1.93; p = 0.057).

**Figure 4:**
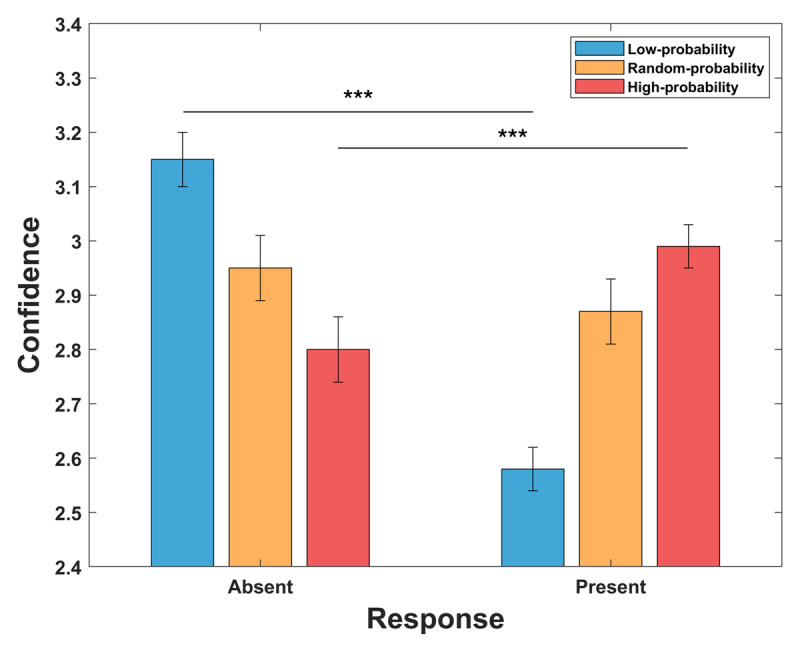
Congruency effect between prior information and response provided. Prior information impacted confidence levels depending on the congruence between target probability and the subsequent response given by the participant. When participants indicated target absence, greater confidence was linked with the low-probability condition (33%) rather than high-probability one (67%). Conversely, when participants reported target presence, higher confidence was associated with the high-probability condition. There was no discernible difference in confidence levels following responses given in the random-probability condition (50%).

We also examined the effect of prior information on confidence when participants reported the presence or the absence of the target (Figure 4). The analyses revealed a significant interaction between prior information and the response provided (F_2,146_ = 104.70; p < 0.001). Post-hoc analyses demonstrated that with the low-probability cue, confidence was higher when participants reported the absence (3.15 ± 0.05) relative to the presence of the stimulus (2.58 ± 0.06; t_74_ = 8.38; p < 0.001), whereas with the high-probability cue confidence was higher for present responses (2.99 ± 0.04) compared to absent responses (2.80 ± 0.06; t_74_ = –3.23; p = 0.002). Conversely, with the random-probability cue, the difference between present and absent responses did not reach significance (present-response = 2.87 ± 0.05; absent-response = 2.95 ± 0.06; t_74_ = 1.59; p = 0.117). Overall, these findings indicate a strong congruency effect: when the presented probability cue matches with the actual stimulus presented or response given by participants, confidence is higher, whereas a mismatch lowers confidence ratings.

### Correlation between prior-dependent modulation of criterion and confidence

**Figure 5:**
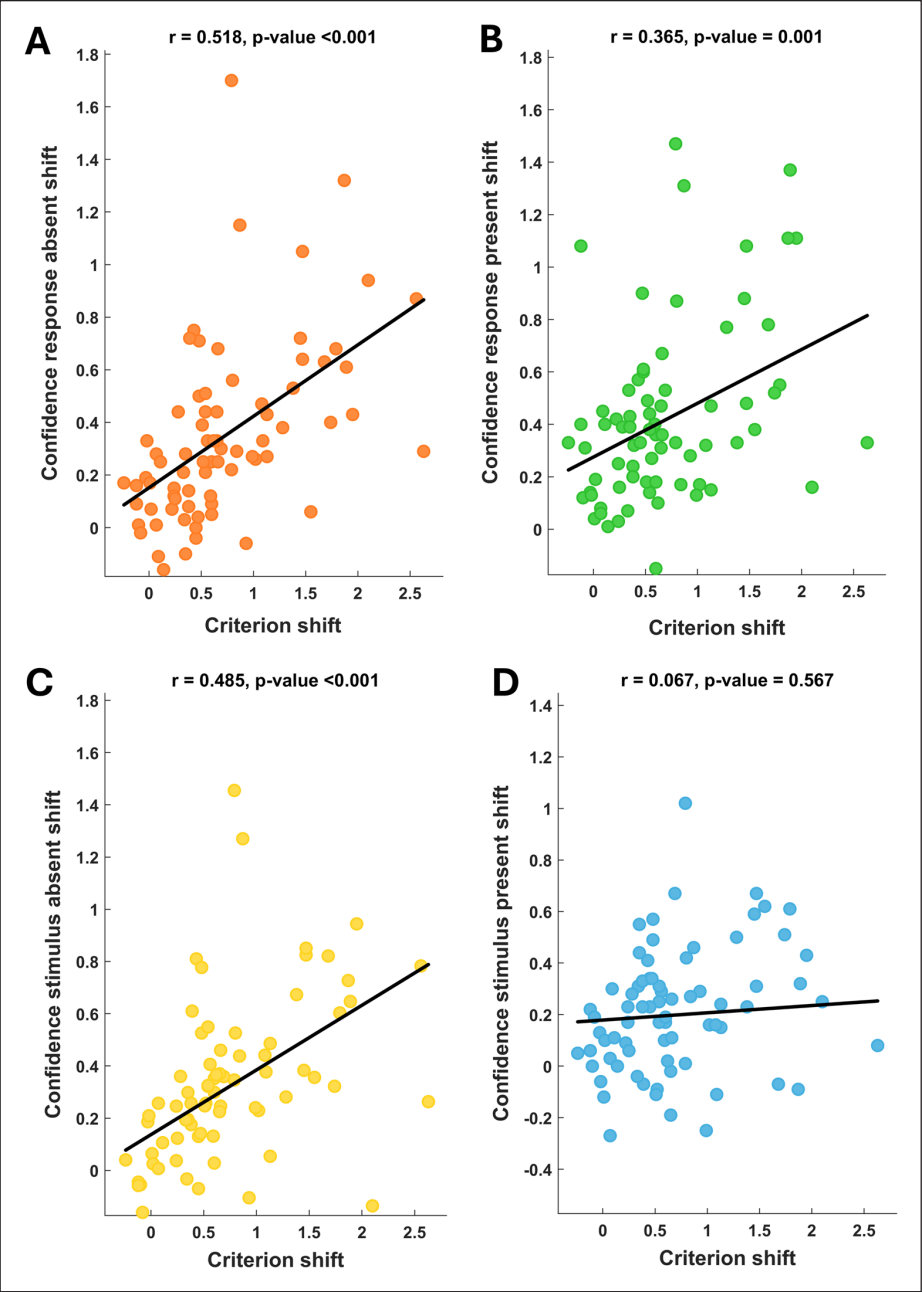
Association between prior-dependent modulation of criterion and confidence. It is observable a significant positive correlation between the prior-dependent adjustment of criterion and confidence, observed in both target-absent (A) and target-present *(B)* responses. Similarly, a positive association driven by prior information was also detected between criterion and confidence adjustment in target-present trials *(C)*, but not in target-absent ones (D).

To investigate the relationship between confidence and criterion, Pearson’s correlation analyses were conducted. The first analysis indicated a significant positive association between the overall criterion and the overall confidence for both low-probability (r_cons_ = 0.28; p = 0.015) and high-probability trials (r_lib_ = 0.26; p = 0.025), but this relationship did not hold true for random-probability trials (r_neu_ = 0.17; p = 0.146). Thus, these results indicated that participants’ overall response bias is associated with the absolute confidence level only when an informative (whether low- or high-probability trial occurrence) but not uninformative prior (random-probability trial occurrence) was presented. Subsequently, we examined whether the prior-dependent modulation of criterion was associated with a corresponding prior-dependent modulation of confidence. The conducted analyses revealed positive correlations between Δ criterion (i.e., how much individuals shift the criterion) and Δ confidence (i.e., how much individuals shift confidence ratings in congruent vs. incongruent conditions) across almost every condition examined (Figure 5). Specifically, there was a significant association between criterion shift and confidence shift for both target-absent responses (r = 0.518; p < 0.001) and target-present responses (r = 0.365; p = 0.001). We found similar results when considering target-absent trials (r = 0.485; p < 0.001). However, this pattern was not found in target-present trials (r = 0.067; p = 0.567). These results suggest a link between criterion modulation and confidence modulation: the more participants shifted their decision criterion according to the presented cue, the more they shifted their confidence ratings in accordance with the given prior.

The absence of correlation in target-present trials might be attributed to participants’ inclination to adopt a conservative criterion due to the task difficulty. Bias toward a conservative strategy is supported by criterion values in random probability trials, demonstrating a positive value (crit_random_ = 0.42 ± 0.05) significantly different from zero (t_74_ = 7.92; p < 0.001. Thus, this general tendency to report the absence of the target could mask the impact of prior information on criterion in trials where the target is indeed present. Crucially, the same trend emerges when considering the provided response: although both correlations are significant, the correlation coefficient for target-present responses is smaller than for target-absent responses.

## Discussion

To investigate the impact that perceptual priors exerted on the level of awareness in decision-making, we recorded the behavioral responses of human observers while performing a probabilistic detection task. Each trial started with the presentation of a prior indicating the likelihood of target’s appearance.

After indicating the target’s presence vs. absence (type 1 decision), participants rated their confidence levels using a 4-level Likert scale (type 2 decision). The theoretical foundation for our observations is grounded in Signal Detection Theory (SDT) and its extensions that incorporate Type 2 judgments (Maniscalco and Lau, 2014). According to this framework, confidence judgments (Type 2 tasks) are thought to emerge from the same internal processes that govern stimulus classification (Type 1 tasks). Stimulus type and response type serve as key moderators in this model because they shape internal signal distributions and decision criteria, thereby influencing both the initial decision (Type 1) and the confidence associated with that decision (Type 2).

When analyzing the SDT indices, the results exhibited a consistent pattern already observed in previous studies (Bang & Rahnev, 2017; Limbach & Corballis, 2016; Tarasi, di Pellegrino, et al., 2022; Tarasi et al., 2024). While sensitivity remained unaffected by priors, a strong impact was found in the decisional bias. Notably, the criterion index was found to be more liberal vs. conservative in the high- vs. low probability trials. Moreover, we revealed a noticeable impact on confidence ratings played by the presented cue, indicating that low probability cues resulted in higher confidence judgments compared to both high and random cues. Despite this strong effect on confidence ratings, metacognitive sensitivity (Meta-d′) remained unaffected by variations in prior probabilities. We further specified this effect, demonstrating that there was greater confidence after the commission of a correct response only in the low probability trials compared to the other conditions. A possible explanation of this result could be attributed to human natural inclination to place criteria in a conservative manner (Rahnev & Denison, 2018). This tendency might lead to individuals having greater confidence in their decisions when operating within a conservative context, because of the alignment of the decision strategy with the individual’s inherent proclivity. Furthermore, given the task’s relative difficulty, a conservative criterion may have been more suitable for the decision-making context, resulting in relatively higher precision in type 2 judgments.

Subsequently, we analyzed whether the congruency between prior and stimulus on the one hand and prior and response on the other had an effect on shaping confidence judgments. In our experimental protocol, congruence occurred when the prior (e.g., high probability of stimulus presence) aligned with the stimulus actually presented (e.g., target presence) or the response provided by the participant (e.g., response present). We unveiled that the congruence effect is a key factor in the generation of confidence judgments. Specifically, when high vs. low probability cues were given, participants exhibited higher confidence for target present vs. absent trials. Similarly, considering given responses, low-probability cues resulted in higher confidence ratings for target-absence reports, while high-probability cues showed the opposite pattern, with higher confidence in target-presence responses. This suggests that participants integrated prior knowledge, sensory information and response outcome when forming their confidence judgments.

Given the strong effect on both criterion and confidence played by prior information, we explored whether there was an association between these two modulations. First, we found a significant relationship between overall criterion and confidence in the high- and low-probability conditions. Instead, when uninformative cues were presented (i.e., random-probability cues), we found no relationship between criterion and confidence. This finding implies that, when a predictive information is not provided, the decision rule applied for type 1 and 2 decision are not equivalent. Therefore, if a participant exhibits overconfidence or underconfidence in the random condition, these inclinations do not imply a liberal vs. conservative type 1 criterion. Furthermore, a significant relationship was observed between criterion shift and confidence shift, emphasizing that the impact of priors on type 1 and 2 judgments results in correlated modulation rates.

Interestingly, correlations based on the response (i.e., present and absent responses) were stronger than correlations related to the stimulus (i.e., present and absent trials). One possible explanation for this variability is that individuals may have more direct awareness of their own responses compared to the stimulus presented, making confidence more directly tied to the response rather than the stimulus itself. This is further confirmed by the lack of correlation for target-present trials while it held when considering the response.

In summary, this study examined the influence of prior information on confidence ratings, offering additional insights into the integration of expectations into human decision-making processes. The findings demonstrated that prior information significantly affects type 2 decisions, with this influence being moderated by a congruency effect between the given prior, the actual stimulus presented, and the provided response. Additionally, we found a correlation between the general Type 1 and Type 2 criteria, as well as a connection in the influence of priors on their modulation rates. Crucially, this relationship held true when an informative prior was provided (and thus the criteria for decision types 1 and 2 could adapt to it), but not in the neutral condition in which prior was uninformative.

Relationship between prior-driven modulations of criterion and confidence could speak to recent proposals of confidence being grounded in self-consistency (Caziot & Mamassian, 2021). Specifically, once participants adjust their decision criterion based on the prior information received (i.e., reporting stimulus presence in high probability trials), they also align their metacognitive bias to maintain internal consistency with their choice, leading to higher confidence. Therefore, criterion modulation could affect confidence through self-consistency, making type 1 and type 2 decision bias strictly connected.

These findings can serve as a starting point for various future explorations. Recent findings suggested that the prior-driven criterion shift is paired by a concurrent modulation of brain oscillatory activity, specifically within the alpha band (8–14 Hz; Tarasi, di Pellegrino, et al., 2022). For this reason, future research could explore whether alpha modulations also underpin confidence shifts or whether other neural signatures are involved in confidence modulation. Additionally, considering the wide variability in prior weighting within the general population (Tarasi et al., 2023; Tarasi, Trajkovic, et al., 2022), with certain individuals more incline to rely on top-down vs. bottom-up signaling (Tarasi et al., 2021; Tarasi et al., 2023; Ursino et al., 2022), it becomes intriguing to explore whether a comparable spectrum of variability extends to the prior-driven confidence shift. These potential investigations could shed light on individual differences in the interplay between prior information and confidence judgments, contributing to a more nuanced understanding of decision-making processes in the general population.

## Data Accessibility Statement

The dataset referenced is accessible to the public through the following OSF link: https://osf.io/jg5yt/.

## Additional file

The additional file for this article can be found as follows:

10.5334/joc.417.s1 Supplemental File 1. Detailed description of the experimental design and correlation analyses.
