# Supplementary material for: Prior Information Shapes Perceptual Confidence
Source: J Cogn. 2025 Jan 7;8(1):11. doi: 10.5334/joc.417 (PMC11736391; doi:10.5334/joc.417)
Supplement: Supplemental File 1. — Detailed description of the experimental design and correlation analyses. [file joc-8-1-417-s1.pdf]

EXPERIMENTAL DESIGN

Visual stimuli for the detection task consisted of 8x8 black-and-white checkerboards appearing in the bottom left visual field which could contain grey circles within each cell (target trials) or not (catch trials). Stimuli were generated using MATLAB and presented on a 18' CRT display (display resolution of 1280 × 1024 pixels, refresh rate 85 Hz) at a distance of ~57 cm in a dimly lit room. Regarding the second phase of the study, which comprised 6 blocks of 90 trials each, each trial began with the presentation of a 1s probability cue presented at the center of the screen followed by a fixation dot. The probability cue was a rectangle with its bottom coloured in red and its top coloured in blue. The percentage of the red shading to the entire rectangle indicated the probability of target presence. There were three levels of cue: high and low cues (informative cues) indicated the probability of the target's presence at 67% and 33%, respectively. Instead, the random cue (un-informative cue) equally predicted (50%) the presence and absence of the target. After a variable delay of 1.2–1.5s, a checkerboard containing (or not) grey circles at the titrated contrast appeared at the bottom left of the monitor for 60 ms. Participants had to determine the presence or absence of the grey circles within the checkerboard and provide a confidence rating for their choice. For the detection task, participants were instructed to indicate the presence or absence of the grey circles within the checkerboard as quickly and as accurately as possible and to press the corresponding keys on the keyboard ('k' with the middle finger and 'm' with the index finger for target-present and target-absent responses, respectively). For the confidence judgment, participants had to rate their confidence in the previous choice on a discrete scale ranging from 1 (low confidence) to 4 (high confidence) using numbers 1-4 on the keyboard. No timeout was set for participants' responses.

CORRELATIONS ANALYSIS

As discussed in the main text, we conducted several correlation analyses to investigate the relationship between criterion and confidence, as well as the relationship between the prior-dependent modulation of the same parameters. First, we computed Pearson correlation between the mean criterion values and the mean confidence ratings, considering both target-present and target-absent trials, as well as present- and absent-responses collectively. We considered the SDT measure *c* as well as the mean confidence ratings

separately for trials preceded by high, low, and random probability cues [mean($crit_{high}$), mean($crit_{low}$), mean($crit_{random}$); mean($conf_{high}$), mean($conf_{low}$), mean($conf_{random}$)].

Subsequently, we investigated the relationship between the prior-dependent modulation of criterion and the prior-dependent modulation of confidence. Regarding criterion, we took the difference between criterion in low probability trials and high probability trials (Δ criterion = $crit_{low} – crit_{high}$). Regarding confidence, we took the difference between confidence ratings in congruent trials and confidence ratings in incongruent trials (Δ confidence). We computed four distinct indices of Δ confidence considering both the stimulus (present or absent) and the response (regarding the presence or absence of the stimulus). In the first case, we calculated the difference between confidence ratings in conservative trials (i.e., when the low probability cue was presented) and liberal trials (i.e., when the high probability cue was presented) separately for target-present trials (i.e., $conf_{high} – conf_{low}$) and target-absent trials (i.e., $conf_{low} – conf_{high}$). Congruent trials are those where the probabilistic cue matches the stimulus (i.e., low probability cue in catch trials or high probability cue in target trials), whereas incongruent trials lack this correspondence. Regarding the response, we calculated the difference between conservative and liberal trials separately for responses indicating the presence (i.e., $conf_{high} – conf_{low}$) and the absence (i.e., $conf_{low} – conf_{high}$) of the target. In this case, in congruent trials the probability cue matches the participant’s response (i.e., low probability cue and stimulus-absent response or high probability cue and stimulus-present response), whereas in incongruent trials there’s no correspondence between the cue and the response.
